# CRM1/XPO1 expression in pancreatic adenocarcinoma correlates with survivin expression and the proliferative activity

**DOI:** 10.18632/oncotarget.25088

**Published:** 2018-04-20

**Authors:** David M. Saulino, Pamela S. Younes, Jennifer M. Bailey, Mamoun Younes

**Affiliations:** ^1^ Department of Pathology and Laboratory Medicine, University of Texas Health Science Center at Houston McGovern Medical School, Houston, TX, USA; ^2^ Department of Medicine, Section of Gastroenterology, Hepatology and Nutrition, University of Texas Health Science Center at Houston McGovern Medical School, Houston, TX, USA

**Keywords:** XPO1, CRM1, biomarkers, pancreas, nuclear export

## Abstract

CRM1/XPO1 (CRM1) is a nuclear export chaperone that mediates the export of proteins essential to growth regulation and tumor suppression. Its overexpression in tumors was found to be associated with poor prognosis. Selective inhibitors of nuclear export are in phase I and II clinical trials for several tumor types. Our aim was to investigate CRM1 expression in pancreatic adenocarcinoma (PAC) and its relationship to survivin expression and the proliferative activity. Sections of tissue microarray containing 76 formalin fixed and paraffin embedded PAC were stained by immunohistochemistry (IHC) for CRM1, survivin, and Cyclin A. Expression levels of CRM1 and survivin and the proliferative activity, the S-phase fraction (SPF) in tumor cells, were determined using a quantitative digital image analysis solution (OTMIAS). Sixty-six of the 76 (86%) PAC showed positive staining for CRM1, and 10 (14%) were completely negative. The mean CRM1 expression levels ranged from 0.3 to 53 units and the median from 0.3 to 45 units. There was significant positive correlation between the mean and median expression levels of CRM1 in tumor cells and the mean and median levels of survivin (p<0.001). Moreover, there was positive correlation between the mean and median CRM1 levels in tumor cells and the SPF (p=0.013). Our results show that CRM1 is expressed in a significant proportion of PAC, and increased CRM1 levels correlates with increased survivin levels and increased proliferative activity.

## INTRODUCTION

Pancreatic adenocarcinoma (PAC) is an aggressive malignancy with a dire prognosis. According to the American Cancer Society, over 53,000 individuals will be diagnosed with pancreatic cancer in 2017 and over 43,000 will die from it [1]. About 40% of patients diagnosed with PAC will present at an advanced stage, resulting in very limited treatment choices and difficult clinical decisions [2]. Even tumors caught relatively early at Stage 1A have an abysmal five-year survival rate of roughly 14%. New treatment methods are thus vital to combating this deadly disease.

Chromosome region maintenance-1 (CRM1), also known as exportin 1 (XPO1), is a nuclear export chaperone that mediates the export of proteins essential to growth regulation and tumor suppression [3]. CRM1 was found to be overexpressed in several types of transformed cells and malignant tissues, and its overexpression correlates with aggressive behavior and poor survival [3]. CRM1 regulates survivin, which is involved in the regulation of mitosis and other important functions related to cell survival. Selective inhibitors of nuclear export are in phase I and II clinical trials for several tumor types [3]. The aim of this study was to 1) determine whether CRM1 is expressed in human PAC and 2) whether this expression correlates with the expression of survivin and tumor proliferative activity as surrogates of CRM1 activity.

## RESULTS

Seventy-six cases with adenocarcinoma were available for evaluation with all three antibodies. Of these cases 29 patients were females and 47 were males with ages ranging from 23-78 years (mean 53, median 52). Twenty-two of the tumors were Grade 1, 30 were Grade 2, and 14 were Grade 3. The grades were not provided on 10 adenocarcinomas. With regard to the pathological stage (pT), 3 of these tumors were pT1, 27 were pT2, 45 were pT3, and 1 was pT4. Sixty-two tumors were node-negative and 14 were node-positive. One case was had distant metastasis (M1).

**Figure 1:**
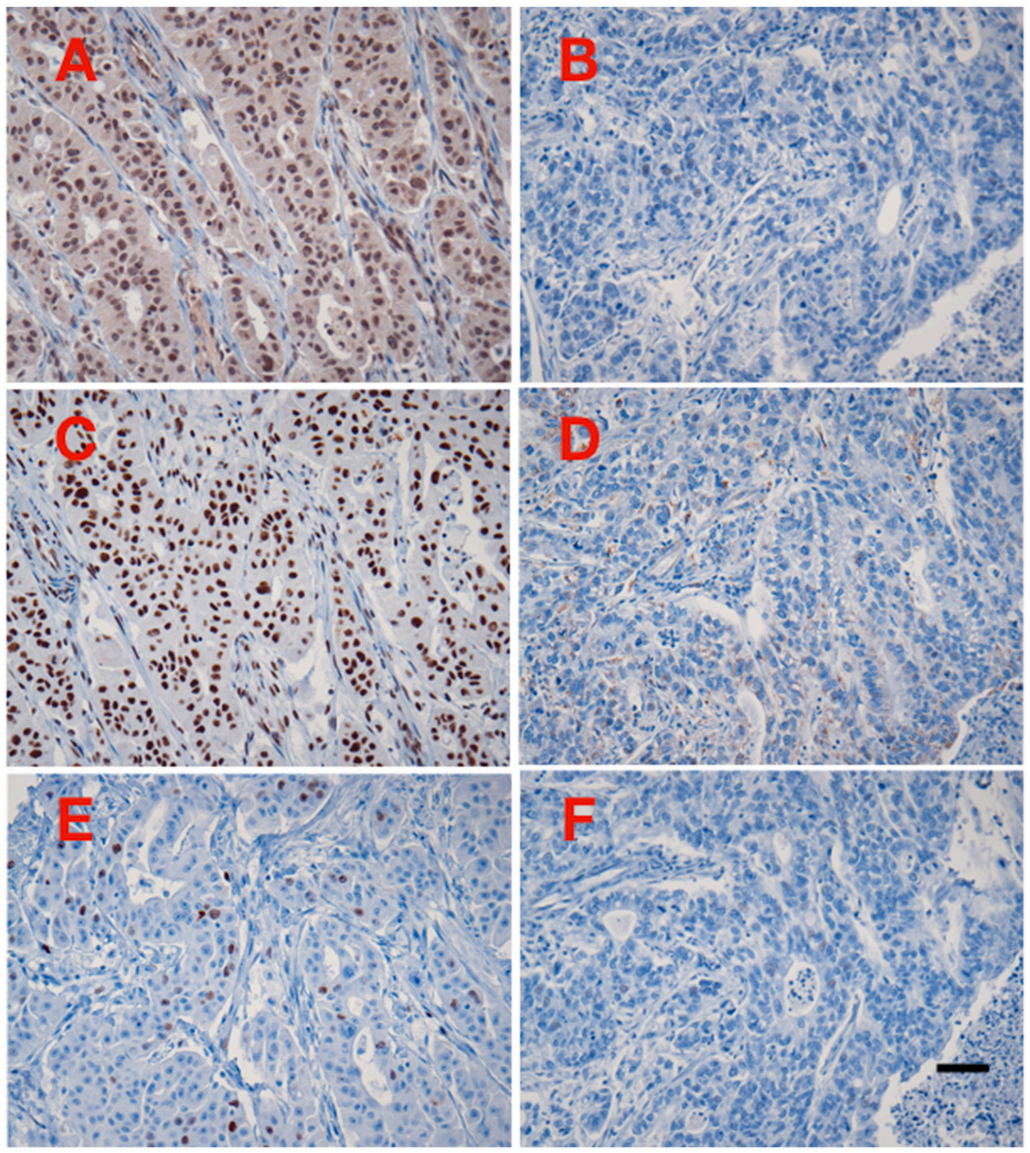
Examples of immunoperoxidase staining for CRM1 **(A and B)**, survivin **(C and D)**, and cyclin A **(E and F)**. A, C, and E represent one case with high CRM1 expression levels, whereas B, C, and D represent one case with negative CRM1 expression. Immunoperoxidas staining with hematoxylin counterstain, 20X microscope objective. Scale bar = 0.1 mm.

**Figure 2:**
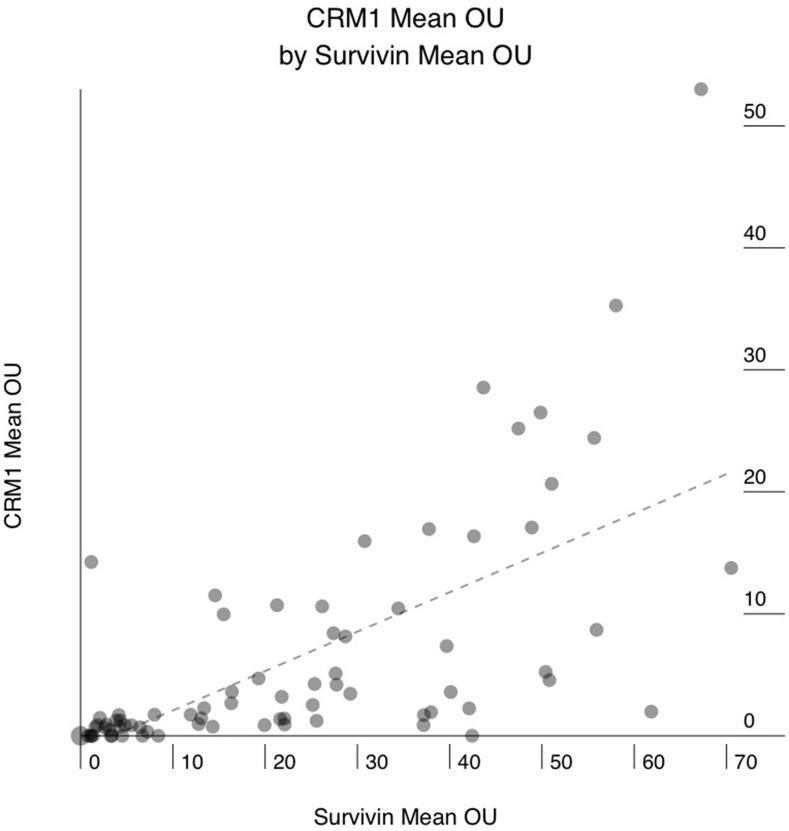
Correlation between mean CRM1 expression in human pancreatic adenocarcinoma (PAC) and mean survivin expression X axis: mean survivin expression in OTMIAS units (OU). Y axis: mean CRM1 expression in OTMIAS units (OU). There is significant positive correlation, p<0.001.

**Figure 3:**
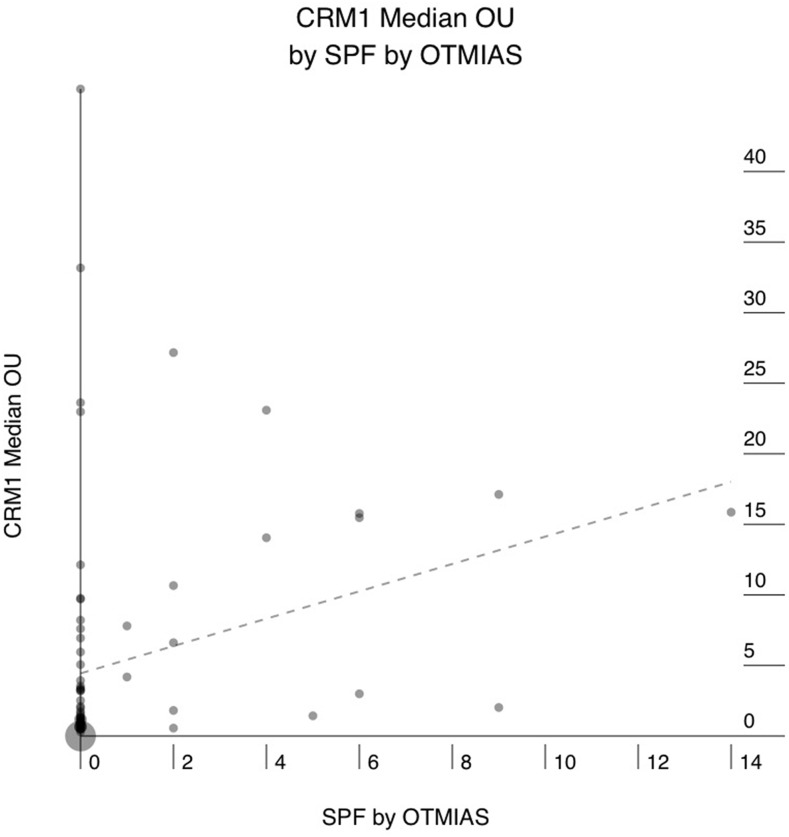
Correlation between median CRM1 expression in human pancreatic adenocarcinoma (PAC) and the S-phase fraction (SPF) as determined by digital image analysis of Cyclin-A stained sections X axis: SPF. Y axis: median CRM1 expression in OTMIAS units (OU). There is significant positive correlation, p=0.013.

Sixty-six of the 76 (86%) PAC showed positive staining for CRM1, and 10 (14%) were completely negative. The mean CRM1 expression levels ranged from 0.3 to 53 Otmias units (OU) and the median from 0.3 to 45 OU. In the positive cases, CRM1 was located in the nucleus with often faint cytoplasmic staining, whereas Survivin stain was only nuclear (Figure 1). There were significant positive correlations between the mean expression levels of CRM1 and survivin (r = 0.653, p < 0.001), the median expression levels of of CRM1 and survivin (r = 0.666, p < 0.001) in tumor cells (Figure 2). Moreover, there were also significant positive correlations between the mean and median expression levels of CRM1 levels and the tumor SPF (r = 0.283, p=0.013; Figure 3).

## DISCUSSION

CRM1 is a nuclear export chaperone responsible for export of cargoes from the cell nucleus to the cytoplasm in eukaryotic cells. While smaller molecules can freely move through the nuclear membrane, larger molecules need an active transport mechanism; a group of proteins called karyopherins, of which CRM1 is a member, aid in this process. Proteins and RNA molecules that need transport display special amino acid sequences called nuclear localization signals. These amino acid sequences are responsible for “tagging” its cargo for the correct destination [4]. Tumors require brisk transport of proteins for growth; CRM1 upregulation can aid in this process. Multiple malignancies were found to be associated with increased CRM1 nuclear export including mesenchymal, hematopoietic, and several epithelial tumors including lung, pancreatic, ovarian, cervical, renal, esophageal, and liver cancers [4]. CRM1 overexpression has been shown to be associated with worse outcomes in many cancer types [5–7]. In some cases, CRM1 upregulation may move key regulatory proteins out of the nucleus such as p53. Normally, p53 proteins are present in the nuclear space to maintain DNA integrity, and movement to the cytoplasm can be devastating [5]. In addition to disrupting protective proteins, CRM1 can also move proteins that aid in cancer growth into the cytoplasm.

The protein survivin is regulated by CRM1, and is believed to exert a cytoprotective effect in the cytoplasm by preventing apoptosis, while in the nucleus it is involved in the regulation of mitosis [5, 8–13]. Survivin has also been linked to poor outcome in various malignancies including PAC [14–18]. Increased survivin expression in PAC was found to be associated with decreased response to chemotherapy [19], and increased serum levels was linked to perineural invasion, venous invasion, lymph node status, differentiation, and relapse [19, 20].

Inhibitors that target CRM1 export pathway have already begun phase I and II clinical trials with encouraging results. In one trial, the first generation CRM1 inhibitor selinexor (KPT-330) has yielded positive results in patients with refractory multiple myeloma [21] and ovarian cancer [22]. In patients with advanced solid tumors, Selinexor treatment achieved stable disease in 17% of patients for greater than 4 months [23]. Additional phase 2 trials with selinexor are underway for refractory multiple myeloma, gynecologic malignancies, recurrent glioblastoma, and head/neck squamous cell carcinomas [24–27]. Second generation CRM1 inhibitors, such as KPT-8602, were recently introduced and shown to be well tolerated and highly active in initial trials [28, 29].

The vast majority of PAC cases in our study showed some degree of CRM1 expression (86%). We also found that the levels of CRM1 expression in PAC correlates significantly with the level of survivin expression and with the tumors proliferative activity as demonstrated by the S-phase fraction. These associations are further indication that increased CRM1 expression in PAC is likely to be associated with increase in its biological activity, since CRM1 regulates survivin expression and both are known to be regulators of cellular proliferative activity.

Because in this study we used a tissue microarray with one core per tumor, we elected not to correlate the expression levels of CRM1 or survivin with tumor stage, nodal status, or grade as the samples in the tissue array may not be representative of the entire tumor. Our correlations between CRM1, survivin, and SPF (determined by image analysis of cyclin A stained sections) is, however, valid because it reflects pared correlations of biomarker expressions in the same cores. For example, the correlation graph between CRM1 and survivin expression levels is based on correlating CRM1 expression in core 1A with survivin expression in the same core 1A, correlating CRM1 in 2A with survivin in 2A, and so forth. This means, in general, that if CRM1 is overexpressed in a core, then survivin is likely to be expressed in same PAC cells in that same core.

Although CRM1 inhibitors were shown to reduce the growth PAC xenografts in mice [30] and to enhance the antitumor activity of Gemcitabine on PAC cell lines [31], there are no human trials yet. In summary, our findings demonstrate that CRM1 is expressed in a significant number of PAC, and suggest that its overexpression is likely to be associated with biologic activity, and should be considered in future clinical trials with selective inhibitors.

## MATERIALS AND METHODS

Glass slides with freshly-cut sections of formalin fixed, paraffin embedded human pancreatic cancer tissue array were obtained from US Biomax (Cat# PA961c, www.biomax.us). The slides came with clinicopathologic information including patient age, sex, tumor type, grade, and stage. The sections were stained by immunohistochemistry (IHC) using antibodies to CRM1 (rabbit polyclonal, Cat# ab24189, Abcam, Inc., www.abcam.com), survivin (rabbit monoclonal antibody, Cat# 2808, Clone 71G4B7, Cell Signaling Technology, www.cellsignal.com), and Cyclin A (mouse monoclonal antibody, Clone 6E6, Abcam). Briefly, following steam heat antigen retrieval in high pH buffer (CRM1 and Survivin), or in a pressure cooker in low pH buffer (cyclin A), sections were incubated for 30 minutes at room temperature with either 1:350 dilution of CRM1 antibody, 1:2000 dilution of Survivin antibody, or 1:200 dilution of the cyclin-A antibody. IHC was carried out utilizing a Dako automated immunostainer (Agilent Technologies, Santa Clara, CA, www.agilent.com) and a Dako EnVision Plus Dual Link peroxidase detection kit (Agilent Technologies). Appropriate positive and negative controls were used.

TIFF images were captured utilizing Olympus BX43 microscope, 20X UPanSApo lens (Olympus Corporation of America, www.olympus-lifescience.com), and Jenoptik ProgRes 7 digital microscope camera. (JENOPTIK Optical Systems GmbH, www.jenoptik.com). The levels of CRM1 and Survivin expression in tumor cells and the S-phase fraction (SPF) were determined using a quantitative digital image analysis solution (OTMIAS, Olive Tree Media, LLC, www.otmedia.com). The expression levels of CRM1 and Survivin are given in OTMIAS Units (OU) which are based on the relative value of expression in each tumor cell compared to similarly processed positive and negative cell line standards. Statistical analysis (Pearson's correlation of paired data) was performed utilizing Wizard statistical software for Mac OS X (Evan Miller, available from the App Store).
